# Disentangling between- and within-person associations of psychological distress and mental well-being: An experience sampling study examining the dual continua model of mental health among university students

**DOI:** 10.1007/s12144-022-02942-1

**Published:** 2022-03-01

**Authors:** Jannis T. Kraiss, Martje Kohlhoff, Peter M. ten Klooster

**Affiliations:** Department of Psychology, Health & Technology, Centre for eHealth and Well-being Research, Faculty of Behavioral, Management and Social Sciences, University of Twente, PO Box 217, 7500 AE Enschede, the Netherlands

**Keywords:** Well-being, Psychological distress, Dual continua model, Between-person, Within-person, Association, Experience sampling

## Abstract

The dual continua model assumes that psychological distress and mental well-being are two related, yet distinct dimensions of mental health. Previous studies did convincingly show the distinctiveness of these two dimensions using mainly cross-sectional research. Despite the importance to distinguish between- and within-person associations in psychological theories, to date, no study specifically distinguished between- and within-person associations for the relationship between distress and well-being. Therefore, the objective of this study was to validate whether the dual continua model actually holds when examined within individuals. Intensive longitudinal data were collected through experience sampling. The sample included 25 university students (mean age = 23.50 years, 56% female), who completed a baseline questionnaire as well as momentary measures of psychological distress and mental well-being three times per day for two weeks. 1,014 timepoints were analyzed using multilevel models and person-mean centering was applied to distinguish between- and within person associations. A significant moderate negative between-person association was found for the relationship between psychological distress and mental well-being (β = −.363, marginal *R*^2^ = 0.15, *p* < .001). The within-person association was also significant and similar in magnitude (β = −.432, marginal *R*^2^ = 0.18, *p* < .001) at the group level. Individual within-person associations between distress and well-being varied substantially, but were negative for almost all participants. This study is an important step towards validating the applicability and universality of this widely used model. The current findings provide preliminary evidence that the dual continua model does not only hold between people, but also on the level it is actually used for, namely within individual people.

It is currently widely recognized that mental health is not merely the absence of psychological symptoms, but also includes the presence of mental well-being. The dual continua model of mental health suggests that psychological distress and mental well-being are two related, yet distinct continua (Keyes, 2005). Psychological distress can be defined as non-specific set of psychological symptoms including, for example, depression, anxiety or stress. Although psychological distress is a multifaceted construct that has been applied to undifferentiated combinations of psychological symptoms, disability and behavioral problems, it is most commonly defined as a state of emotional suffering characterized by symptoms of depression and anxiety (Drapeau et al., 2012; Viertiö et al., 2021). In the context of mental well-being, two components of well-being can be distinguished. One is subjective (or hedonic) well-being, which involves experiencing positive emotions and being satisfied with life (Diener & Ryan, 2009; Diener et al., 1999). The other one is defined as psychological (or eudaimonic) well-being and includes dimensions such as self-acceptance, purpose in life or autonomy (Ryff, 1989, 2014; Ryff & Keyes, 1995).

The distinctiveness or discriminant validity of psychological distress and mental well-being has been extensively demonstrated for different age groups, settings and cultural contexts using correlational and confirmatory factor analytic techniques (e.g., De Vos et al., 2018; Franken et al., 2018; Grant et al., 2013; Keyes, 2005; Keyes et al., 2008; Trompetter et al., 2017). These studies generally demonstrated moderate correlations between well-being and measures of psychopathology and consistent superior fit of a correlated bipolar (2-factor) model over a unipolar (1-factor) model of mental health (Iasiello & Van Agteren, 2020). Also, quite a large body of research examined the longitudinal relationship between mental well-being and psychological distress. Those studies suggest that the presence of mental well-being reduces the future risk of experiencing psychological problems (Lamers et al., 2015) or that the absence of mental well-being independently increases the risk of future depression (Schotanus-Dijkstra et al., 2017b; Wood & Joseph, 2010) and even mortality (Huppert & Whittington, 2003). Other studies found that mental well-being is an important predictor for recovery from psychological issues (Iasiello et al., 2019; Schotanus-Dijkstra et al., 2019).

While these previous studies offer convincing evidence for a degree of independence of mental well-being and psychological distress, all studies were either cross-sectional or longitudinal with only a small number of measurement points. This limits our understanding of the true nature of the relationship between psychological distress and mental well-being, in terms of the level at which the discriminant validity of the two continua actually holds. Cross-sectional studies, such as the previous CFA studies, can only establish so-called *between-person* associations. Between-person analyses can be used to examine, for example, whether people with more psychological distress than others also show higher levels of mental well-being. However, cross-sectional data cannot answer questions related to *within-person* processes (Curran & Bauer, 2011; Wang & Maxwell, 2015). When examining within-person associations, the variability around means of individuals is analyzed (Hamaker et al., 2007). For instance, within-person associations might be used to investigate whether a person who reports higher mental well-being than usual also reports a higher level of psychological distress at the same time point. By definition, cross-sectional studies cannot be used to study within-person associations, since they only contain one observation per participant, making it impossible to study variability around individual means. Longitudinal data do provide the opportunity to identify both relationships that hold within persons as well as relationships that hold across persons, but this requires careful specification of both effects using multilevel modelling (Curran & Bauer, 2011; Hoffman, 2007; Hoffman & Stawski, 2009; Wang & Maxwell, 2015). Without statistically seperating these effects, longitudinal associations provide an ambiguous and difficult to interpret blend of both between- and within-person effects (Curran & Bauer, 2011; Wang & Maxwell, 2015).

It has been convincingly argued that, especially in psychology, it is important to distinguish between-person and within-person associations (Curran & Bauer, 2011; Hamaker, 2012). The reason for this is that most psychological theories or models aim to make inferences about associations or mechanisms that take place within people (Curran & Bauer, 2011; Hamaker, 2012; Hoffman & Stawski, 2009). In this context, the assumption usually is that increasing or decreasing one variable leads to an increase or decrease in another variable within individuals. Although the dual continua model holds that mental well-being and mental illness are related yet distinct phenomena, concepts or dimensions, neither Keyes nor other proponents of the dual continua model explicitly state whether it specifically applies to either the between-person or within-person level or both. Nonetheless, the model is increasingly used as a framework for so-called positive psychological interventions, usually assuming that these interventions aimed at enhancing well-being components such as positive emotions, optimism and positive relations can positively impact both continua of mental health (Iasiello & Van Agteren, 2020; Schotanus-Dijkstra et al., 2017a; Westerhof, 2016). As with other psychological models, therefore, implications of the dual continua model are drawn for processes within people, even though most empirical studies have analyzed associations on a between-person level only. However, strict assumptions of statistical *ergodicity* must be met in order to generalize findings from the between-person to the within-person level. For a process to be ergodic, it needs to be homogenous across individuals and stable across time (Molenaar, 2004). It has clearly been shown that this assumption is rarely met, and that between-person and within-person associations can substantially differ in magnitude or even in direction (Curran & Bauer, 2011; Hamaker, 2012; Hoffman & Stawski, 2009; Kievit et al., 2013; Van de Pol & Wright, 2009; Wang & Maxwell, 2015). This calls for designs and analyses that clearly distinguish these two sources of information.

In order to examine associations within individuals, repeated measures data needs to be utilized (Collins, 2006; Molenaar & Newell, 2010; Molenaar, 2004; Raudenbush, 2001), and ideally this data should be intensive longitudinal data containing a relatively high number of observations per participant (Hamaker et al., 2018). One way to collect such intensive longitudinal data is experience sampling, also known as ecological momentary assessment (Larson & Csikszentmihalyi, 2014). Experience sampling is a research procedure that involves repeated sampling of participants’ current behaviors, feelings or thoughts in real time in their natural environment (Shiffman et al., 2008). Using experience sampling has several potential advantages, including the reduction of retrospective memory bias and increased ecological validity (Scollon et al., 2003; Versluis et al., 2021). Experience sampling has been widely used in several research fields, including studies of emotion regulation (e.g. Ebner-Priemer et al., 2009) or affect dynamics (Ebner-Priemer & Trull, 2009; Hamaker et al., 2015; Koval & Kuppens, 2012) and to specifically distinguish between- and within-person associations (Senker et al., 2020).

Despite the relevance of distinguishing between- and within-person associations, previous studies on the dual continua model exclusively focused on the association of psychological distress and mental well-being on a between-person only or mixed level. To our knowledge, no previous study specifically disaggregated between- and within-person associations of psychological distress and mental well-being. Although it is reasonably conceivable that, over a certain time period, a person can experience both higher well-being and higher distress on average than others, it may be more difficult to imagine that an individual can truly experience both high well-being and high distress at the same time. To date, however, it remains unclear whether momentary feelings of well-being and distress are also sufficiently distinct within persons over time.

Therefore, the objective of this study is to examine the relationship between psychological distress and mental well-being while explicitly disaggregating between- and within-person associations. For this purpose, we will utilize intensive longitudinal data collected in daily life among university students about daily psychological distress and mental well-being. For the discriminant validity of the two continua of mental health to hold at both the between-person and within-person level, similar significant – yet not too strong – negative associations between distress and well-being are expected at both levels of analysis. If the within-person associations are substantially different compared to between-person associations, this would have relevant implications for the use of the model for both research and intervention practice. Considering that associations on the between- and within-person level can substantially differ (Curran & Bauer, 2011; Hamaker, 2012), this is an essential step towards validating the universality and applicability of this widely used model.

## Method

### Participants

A convenience sample of 34 students was recruited by two psychology students from their own personal network as part of their qualification for their bachelor theses. Inclusion criteria for the study were: 1) availability of an Android or iOS smartphone connected to the internet; 2) sufficient level of the English language; and 3) currently being enrolled in university. Power analyses for determining the required sample size needed to have sufficient statistical power at both the within-person (level 1) and between-person level (level 2) is a complex and yet unresolved issue for ESM studies (Gabriel et al., 2019). We therefore aimed to include at minimum 23 participants to be able to detect at least a moderate between-person association (*r* = .50) between psychological distress and mental well-being with 80% power (α = .05, one-sided). Although we did not a-priori know the exact power of our study to examine within-person effects, this sample size corresponds roughly to the median number of 19 participants in the ESM studies reviewed by Van Berkel et al. (2017). The study protocol was approved by the Ethics Committee of the Faculty of Behavioural, Management and Social Sciences of the University of Twente (#191314) and all participants provided active informed consent within the ESM application.

### ESM Protocol

The Ethica Data platform (https://ethicadata.com/) and associated smartphone application were used to design the ESM study protocol and to collect the data. As typical for ESM studies (Dejonckheere & Erbas, 2021; Yearick, 2017), the study consisted of an extensive baseline survey, assessing sociodemographic characteristics and trait-like questionnaires for the constructs of interest, and multiple short questionnaires assessing momentary state assessments of the constructs of interest and contextual variables each day for a period of 14 days. The data was collected from April 6th to April 19th, 2020 and all participants started the study on the same day. ESM studies involving multiple assessments per day typically run from three days to three weeks (Conner & Lehman, 2012). Van Berkel et al. (2017) reported a median duration of two weeks in typical ESM studies, whereas Yearick (2017) found a median duration of 8 days in her review.

Participants completed both the baseline survey and the daily assessments in the Ethica app on their own smartphone. The baseline questionnaire was triggered in the morning on the first day of the study and did not expire for the remainder of the study. Interval-contingent sampling (Conner & Lehman, 2012; Wheeler & Reis, 1991) was used for the momentary assessments in which participants received a push notification to complete the questions at set times in regular intervals throughout each day at morning (10 a.m.), afternoon (3 p.m.) and evening (8 p.m.). Participants that did not complete the assessment received one reminder after 90 min and after 3 h the questions expired. Interval-contingent triggering is the most common sampling strategy used in ESM studies (Van Berkel et al., 2017; Yearick, 2017), and the amount of surveys in typical ESM studies averages around three per day.

### Measures

The study consisted of two surveys, a baseline survey to obtain trait-level measures of mental well-being and distress and a three-daily survey assessing momentary state-level assessments of the same constructs. All materials in the current study were administered in English.

#### Trait Measurements

Trait psychological distress was assessed with the 14-item Hospital Anxiety and Depression Scale (HADS; Zigmond & Snaith, 1983), which measures the presence of mild forms of anxiety and depression in the past week. Item are scored on a Likert scale from 0 (*not at all*) to 3 (*very often*), with higher mean scores being indicative of increased depression or anxiety, respectively (range 0–3). Based on 1000 bootstraps, Cronbach’s alpha was estimated to be .70 for the total score of the HADS (95% CI = 0.51 to 0.81), and .79 (95% CI = 0.61 to 0.88) and .67 (95% CI = 0.30 to 0.83) for the anxiety and depression subscales, respectively. Trait mental well-being was assessed with the Short Warwick-Edinburgh Mental Well-being Scale (Stewart-Brown et al., 2009; Vaingankar et al., 2017). The SWEMBS includes 7 items about thoughts and feelings related to mental well-being over the last week (e.g., to what extent people feel optimistic, or feel relaxed). Items are scored on a Likert scale ranging from 0 (*none of the time*) to 5 (*all of the time*). Higher total scores indicate higher mental well-being (range 0–35). The SWEMBS primarily measures eudaimonic well-being, but also assesses hedonic well-being (Fat et al., 2017; Haver et al., 2015). Bootstrapped Cronbach’s alpha was .61 for the SWEMBS (95% CI = 0.31 to 0.79).

#### State Measurements

State psychological distress was measured with two single-item visual analogue scales (VAS) ranging from 0 (*not at all*) to 100 (*extremely*). One item asked about feelings of depression (“To what extent do you feel down right now?”), while the other question was about feelings of anxiety (“How anxious do you feel right now?”). The mean score of both items was used to represent one overall psychological distress score at each timepoint. State mental well-being was measured with the SWEMBS as well (Stewart-Brown et al., 2009; Vaingankar et al., 2017). For this purpose the recall period in the instruction was changed to refer to the past 2 h.

The SWEMBS was used because it is a well-validated and relatively short measure of well-being. Furthermore, to our knowledge, there are no appropriate single-item scales for well-being. These items than usually assess well-being in a limited way, for example by solely asking about happiness (Griffiths & Stefanovski, 2019; Stieger et al., 2021) or including positive affect items. However, we were interested in a more comprehensive assessment of well-being, also including eudaimonic components of well-being. Visual analogous scales were used for the other questions, as they have some advantages compared to discrete response scales, including higher precision and more variability in responses (Studer, 2012). Single-item VAS scales have also frequently been used in psychological studies to make assessments (Huang et al., 2020; Stieger et al., 2021; Williams et al., 2010) and VAS scales are also used in experience sampling research (e.g., Maes et al., 2015; Nisenbaum et al., 2010; Palmier-Claus et al., 2019). We decided to aggregate the anxiety and depression measures, since previous studies validating the two continua model also often use general symptom measures to operationalize mental illness and not specific ones such as depression or anxiety measures (e.g., Franken et al., 2018; Iasiello et al., 2019; Lamers et al., 2015; Westerhof & Keyes, 2010). Therefore, aggregating depression and anxiety in the current study better resembles how the dual continua model is applied in the literature and how it has been validated before. Furthermore, we aimed to increase the reliability of the latent psychological distress scores by using two instead of only one effect indicators.

To determine the internal consistency of the state measurements, an approach based on generalization theory outlined by Bolger and Laurenceau (2013) was used. For this approach, a random effects ANOVA with item scores as dependent variable and random effects for person, time and item and higher order interactions of these effects was specified. The variance components from this model can then be used to determine whether within-person change is assessed reliably (Bolger & Laurenceau, 2013). Using this method, we found a reliability of 0.82 and 0.70 for the SWEMBS and distress measure, suggesting that within-person change can be measured reliably with the state measures.

### Data Analyses

All analyses were conducted in R (R Core Team, 2020). Multilevel mixed models were used to analyze the data, which can adequately handle missing values and the nested structure of longitudinal data. The nlme package was used to run mixed models (Pinheiro et al., 2020), and ggplot2 was used to visualize between- and within-person associations. From the total 34 recruited students, three participants were excluded from the analysis because they did not fill in the baseline questionnaire. In line with common ESM practice (Conner & Lehman, 2012), 6 additional participants who completed less than 50% of the daily assessments were also excluded from the analysis. The remaining dataset included 1014 timepoints, of which 110 rows contained missing values (10.8%). Validity of the state measures was examined using two separate multilevel models with the average baseline scores for distress or mental well-being as fixed effects and momentary observed distress or mental well-being from the first week of measurements as dependent variable. A strong positive association was found between baseline and momentary mental well-being (β = .52, *p* < .001), and a weak borderline significant association between baseline and momentary psychological distress (β = .24, *p* = .098).

To disaggregate between- and within-person associations, the traditional strategy of person-mean centering in combination with multilevel modelling was used (Bolger & Laurenceau, 2013; Curran & Bauer, 2011; Van de Pol & Wright, 2009; Wang & Maxwell, 2015). This was done as follows: one average score of mental well-being across all observations was calculated for each participant, resulting in a person mean for each participant. The person mean was then subtracted from the momentary mental well-being score at each observation, resulting in person-mean centered scores for each time point. This variable reflects the variability of each person around their own mean and can be used to examine within-person associations. Person-mean centering eliminates all between-person variability, making it possible to effectively disaggregate between- and within-person variance (Curran & Bauer, 2011; Hamaker, 2012; Hamaker et al., 2007).

In the first model, time-varying state well-being was entered as fixed covariate and time-varying state psychological distress was entered as dependent variable. The resulting regression coefficient in this model represents a weighted average of both between- and within-person association, as it purely reflects the associations of well-being and distress across all observations, without distinguishing variability between and within subjects. In the second model, time-invariant person-mean and time-varying person-mean centered scores were entered simultaneously as fixed covariates in the model, and time-varying observed psychological distress was again entered as dependent variable. This model distinguishes between- and within-person associations, with the resulting regression estimate of the person mean representing the between-person association, while the estimate of the person-mean centered score represents the within-person association. Both models were additionally run with timepoint included as additional fixed covariate, to examine whether coefficients obtained from the models substantially differ when controlling for a potential effect of time (Bolger & Laurenceau, 2013). To determine whether aggregating state anxiety and depression into one composite measure of psychological distress substantially changes the conclusions drawn from the analyses, we conducted additional sensitivity analyses with anxiety and depression measures as dependent variables separately.

All models were two-level models, with observations (level 1) nested within participants (level 2). Restricted maximum likelihood and a first-order autoregressive (AR1) covariance matrix was used. This covariance structure was chosen based on the assumption that correlations between measurements decline exponentially over time, and because AR1 showed a significantly better fit versus a model with a variance components or compound symmetry structure according to the log-likelihood ratio tests (*p* < .001). The models included random effects for participants’ intercepts and slopes. To obtain standardized regression coefficients (β) next to non-standardized regression estimates, the dependent variable and all variables that were used as fixed covariates were additionally converted to Z-scores. The strength of standardized estimates was interpreted as small (β > .10), medium (β > .30) and large (β > .50) (Cohen, 1988). Since standardizing in multilevel models is problematic and remains a topic of debate (Schuurman et al., 2016; Wang et al., 2019), we additionally obtained marginal and conditional R^2^ values from each model (Nakagawa & Schielzeth, 2013). Given the consistent moderate to strong negative association between measures of distress and well-being reported in previous research, one-sided *p* values were used for testing both the aggregated and disaggregated associations between mental well-being and distress in the different models.

## Results

### Sample Characteristics

The final sample (Table 1) comprised 25 students (56% female) with a mean age of 23.50 (*SD* = 2.82) years. Most of the students had the German nationality (88%) and studied a subject in the field of the social sciences (72%). Baseline scores of the SWEMBS (*M* = 24.28, *SD* = 2.79) indicated that the sample had an overall score of mental well-being which was comparable to overall scores in the same age group obtained in a previous National Health Survey (Fat et al., 2017). Scores on the depression and anxiety subscale of the HADS were somewhat higher compared to previous surveys among people in the same age group (Jörngården et al., 2006). On average, participants completed 36.2 momentary state questionnaires (range 22–42).

**Table 1:** Baseline characteristics of the included sample (*N* = 25)

Variable	*M*	*SD*	*n*	%
Age	23.50	2.82	–	–
Gender				
Female Male	- -	- -	14 11	56 44
Nationality				
German Australian Other	- - -	- - -	11 1 2	44 4 8
Field of study				
Social sciences Natural sciences Arts Other/not applicable	- - - -	- - - -	18 1 5 15	72 4 20 60
Highest degree				
High school Bachelor	- -	- -	15 10	60 40
SWEMBS	24.28	2.79	–	–
HADS-D HADS-A	4.40 7.08	2.47 3.23	- -	- -

*HADS-A* Hospital Anxiety and Depression Scale-Anxiety subscale, *HADS-D* Hospital Anxiety and Depression Scale-Depression subscale, *SWEMBS* Short Warwick-Edinburgh Mental Wellbeing Scale

### Aggregated Associations between Mental Well-Being and Psychological Distress

**Fig. 1:**
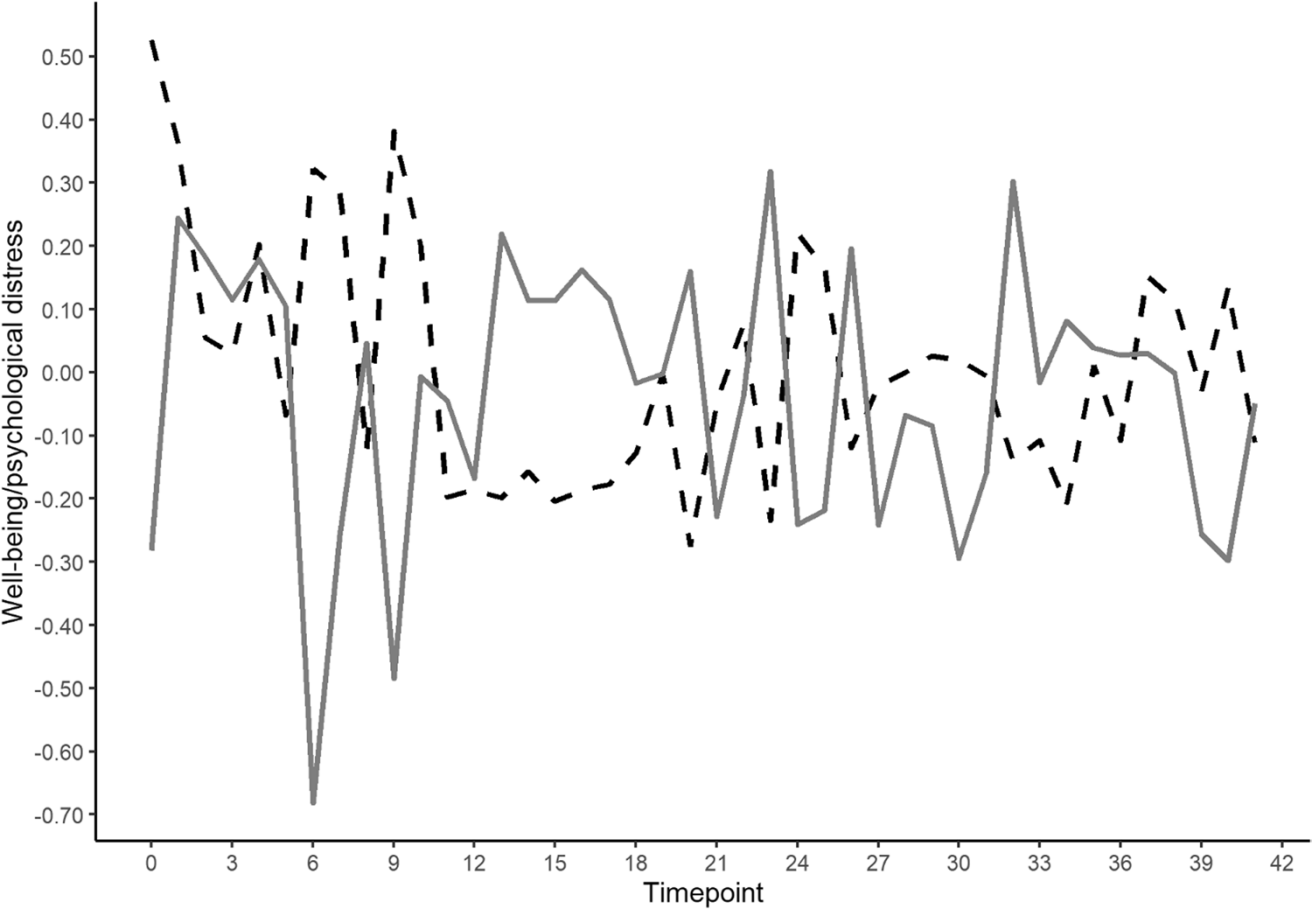
Estimated marginal means of reported psychological distress (dashed black line) and mental well-being (solid gray line) per measurement point (z-score standardized)

Figure 1 shows standardized psychological distress and mental well-being scores across all participants for all timepoints. Visual inspection shows quite a strong variability in both distress and well-being over time. The plot also suggests that there is a negative, but imperfect, association between distress and well-being, with distress scores generally being higher in moments when well-being sores are comparably low. In the blended model that does not distinguish between- and within-person associations, a strong significant overall association between well-being and psychological distress was found (β = −.52, marginal *R*^2^ = 0.25, *p* (one-sided) < .001). When including time as additional fixed covariate in the model, time was significantly associated with psychological distress (*p* = .02), but the overall association between mental well-being and distress did not substantially change (β = −.52, marginal *R*^2^ = 0.26, *p* (one-sided) < .001).

### Disaggregated between and within-Person Associations

The analyses that distinguish between- and within-person associations showed that mental well-being and distress were moderately significantly correlated between people (β = −.36, marginal *R*^2^ = .15, *p* (one-sided) < .01). A somewhat stronger, but still moderate association was found between psychological distress and mental well-being within people (β = −.43, marginal *R*^2^ = .18, *p* (one-sided) < .001) at the group level. Table 2 summarizes the findings from all models.

**Table 2:** Overall associations between time-varying mental well-being and psychological distress (Model 1) and disaggregated between- and within-person associations between mental well-being and psychological distress (Model 2)

Model	Predictor	Estimate (95% CI)	Standardized estimate (95% CI)	Marginal *R*^2^	*F*-value (*df*)	*p* value
1	Well-being	−1.93 (−2.401 to −1.47)	−.52 (−.64 to −.39)	0.25	65.74 (878)	< .001
2	Well-being PM	−2.36 (−3.80 to −0.92)	−.36 (−.58 to −.14)	0.15	11.40 (23)	.003
	Well-being PMC	−1.93 (−2.42 to −1.44)	−.43 (−.54 to −.32)	0.18	59.45 (878)	< .001

*CI* confidence interval, *df* Degrees of freedom, *PM* Person-mean, *PMC* Person-mean centered, Model 1 = Time-variant observed psychological distress is the dependent variable, time-varying observed mental well-being is included as fixed covariate. This model does not clearly disaggregate between- and within-person associations. Model 2 = Time-variant observed psychological distress is entered as dependent variable, person-mean and person-mean centered are entered simultaneously as fixed covariates. This model clearly disaggregates between- and within-person associations between psychological distress and mental well-being. In this model, the effect of the person-mean represents the between-person association, the effect of the person-mean centered variable represents the within-person association. One-sided p-values are reported in this table

Again, time was positively associated with distress in the disaggregated model (*p* = .02), but the coefficients did not significantly change, neither for the between (β = −.36, marginal *R*^2^ = .16, *p* (one-sided) < .01) nor for the within-person association (β = −.43, marginal *R*^2^ = .18, *p* (one-sided) < .001).

**Fig. 2:**
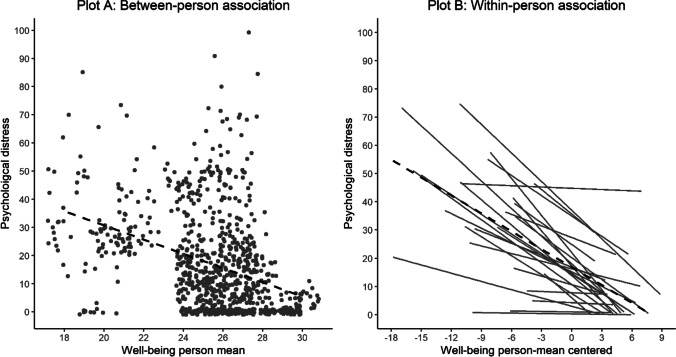
Between-person (Plot A) and within-person association between psychological distress and mental well-being. Note. The dashed lines represent the overall regression line (fixed effect) of the between- and within-person association. In Plot B, the solid black lines indicate individual slopes for each participant for the within-person association between distress and well-being

Fig. 2 visualizes the association between distress and well-being between and within individuals. Although results from the models indicate that at the group level the association is similar between and within people (in magnitude), the right plot (panel B) of this figure shows considerable inter-individual variability in the magnitude of this association. Most of the individual slopes indicate that there is a negative (or at least not positive) association within individuals. However, there are also individuals for which the association does seem to be substantially different compared to the overall within- and between-person association (i.e., stronger or weaker). This suggests some degree of universality of the association between well-being and distress, but also illustrates that the association is not equally present within each individual.

### Sensitivity Analyses

The sensitivity analyses with anxiety and depression separately as dependent variables showed similar results. The aggregated association between mental well-being and anxiety was significant (β = −.35, marginal *R*^2^ = .13, *p* (one-sided) < .001), and so was the between-person (β = −.28, marginal R^2^ = .12, *p* (one-sided) = .02) and within-person association (β = −.28, marginal *R*^2^ = .07, *p* (one-sided) < .001). For the relationship between depression and well-being, a significant aggregated (β = −.55, marginal *R*^2^ = .32, *p* (one-sided) < .001) as well as between-person (β = −.20, marginal *R*^2^ = .04, *p* (one-sided) = .01) and within-person association (β = −.48, marginal *R*^2^ = .23, *p* (one-sided) < .001) was found.

## Discussion

The objective of this study was to examine the association between psychological distress and mental well-being and explicitly distinguish between- and within-person associations of these constructs. By doing so, we aimed to further validate the universality and applicability of the widely used dual continua model of mental health (Keyes, 2002; Keyes, 2005), assuming discriminant validity of psychological distress and mental well-being (Iasiello & Van Agteren, 2020). For this purpose, intensive longitudinal data collected in daily life through experience sampling was utilized and analyses were performed that can distinguish between- and within-person associations (Curran & Bauer, 2011; Hamaker, 2012).

In the model that explicitly disaggregates between- and within-person associations, we found a moderate negative association of distress and well-being between and within individuals. The overall association was comparable in magnitude on both levels of analysis. The significant between-person association suggests that people with higher distress than others have lower well-being on average. The overall strength we found for this association was relatively comparable to previous studies examining the association between distress and well-being (e.g., Grant et al., 2013; Keyes, 2005). In this context, a considerable amount of prior research examining the dual continua model between persons provides evidence that distress and mental well-being are related, yet distinct dimensions of mental health (Franken et al., 2018; Iasiello & Van Agteren, 2020; Keyes, 2005; Keyes et al., 2008). The fact that we also found that distress and well-being were related between persons, but not strongly enough to suggest that they represent two sides of the same continuum, further supports those previous studies and the notion that distress and well-being rather represent two distinct dimensions of mental health (Keyes, 2005).

We also found a moderate negative association between distress and well-being within people. This negative within-person association indicates that when a person scores higher in psychological distress than his or her own average, this person also tends to experience lower well-being in that moment. This association therefore allows to unambiguously draw conclusions about the relation of distress and well-being within people, as it eliminates all between-person variance and solely examines variability that occurs around means of individuals (Curran & Bauer, 2011; Hamaker, 2012; Hamaker et al., 2007). This is relevant, as it has been convincingly argued that associations found on a between-person level can only be generalized to processes occurring within individuals under strict assumptions of ergodicity (Curran & Bauer, 2011; Hamaker, 2012; Hoffman, 2007). At the group level, the magnitude of the within-person association was similar to the correlation found between people in the current and previous studies (Grant et al., 2013; Keyes et al., 2008). This suggests that psychological distress and well-being are also moderately related within people, but the strength of the association was also not strong enough to assume that distress and well-being are two completely distinct ends of one continuum (Keyes, 2005). Therefore, these findings offer some first evidence that the dual continua model also seems to hold when examined within people. This not only increases our knowledge on the relationship between psychological distress and well-being, but also further increases the applicability and universality of this widely used model. One specific finding to discuss in this context is that we did not find a significant correlation between trait and state measures of psychological distress. While this could be a sign for questionable validity of the state measures, it could also simply come from the fact that the trait and state measures for psychological distress were rather different in their nature and content. Furthermore, correlations between traits and aggregated states from repeated measurements should not alone be considered to judge convergent validity of an ESM measure (Rauthmann et al., 2019). The sensitivity analyses additionally showed that the conclusion that can be drawn from the analyses did not change when anxiety and depression were analyzed separately, since well-being was significantly – but not too strongly – related with both anxiety and depression. This suggests that the two-continua model also holds when examining specific dimension of distress, instead of one overall score.

Interestingly, we did find substantial inter-individual variability in the association between psychological distress and well-being. Within some people this association seemed not to be present at all, while for some people it seemed considerably stronger compared with the overall association. This suggests that the assumption of the dual continua model might not necessarily hold for each individual. Psychological distress and well-being appear rather the same for those people showing a relatively strong correlation. In those cases, mental health might rather be described as two opposite ends of the same continuum. Another explanation for this inter-individual variability could be that the idea people have about well-being and distress differs quite substantially, which might affect their responses to the questionnaires. It could for example be that some people think of mental well-being as the absence of symptoms, while others do not. It could also be that well-being measured with the SWEMBS showed less variability than psychological distress measured with VAS scales, which could have suppressed the correlations, at least for some of the participants. Nonetheless, these findings could have important implications for applied research and interventions aimed at improving mental health, as this would mean that for some individuals distress and well-being would not need to be considered distinct outcomes of treatment as previously suggested (e.g., De Vos et al., 2018; Franken et al., 2018; Trompetter et al., 2017).

### Implications and Future Directions

Our findings provide preliminary evidence that psychological distress and well-being are only moderately associated when specifically examining this association within individuals. This suggests that the widely used dual continua model of mental health does not only hold when examined between people, but also when examined within people. This is relevant for (clinical) practice, as it indicates that interventions should focus on both ameliorating clinical symptoms but also promoting positive capacities. However, for future studies it might be interesting to further examine the difference in between- and within-person associations in different groups or (cultural) contexts to see whether these conclusions also can be generalized to other groups. Another important implication is that we found that the magnitude of the relationship between distress and well-being seems to substantially vary for different people. This suggests that the assumption of the dual continua model does not necessarily hold for each individual. This could have important implications for treatment, since this could mean that distress and well-being do not necessarily have to be considered different outcomes of interventions for all people. It could also be interesting to explore prognostic factors influencing the relationship between psychological distress and well-being. This could help to determine why distress and well-being are so strongly related for some people, while this seems not to be the case for other people. It could be, for example, that people in whom this association is not that strong, tend to have more resources protecting their well-being from the impact of distress. One example of potential factors that could be examined is this context are emotion regulation strategies (Gross, 1998; Kraiss et al., 2020).

### Limitations

Some limitations should be considered when interpreting the current findings. First, the sample was limited to a mainly German university student population from one specific university. Although experience sampling studies generally do not aim to be representative, this limits generalizability of the current findings and the sampling strategy might have increased the chance for selection bias (Etikan et al., 2016). The fixed sampling scheme we used has the advantage of being more convenient and less interfering for participants, which is likely to increase compliance. However, compared to a random sampling scheme, it potentially leads to increased reactivity and less ecological validity, since questionnaires are only triggered during specific moments of the day (Conner & Lehman, 2012; Dejonckheere & Erbas, 2021). Second, despite the collection of intensive longitudinal data, only correlational conclusions can be drawn from the current analyses, while no conclusions about causality or temporal precedence can be made. Third, data for this study was collected during the first COVID-19 lockdown in April 2020. Research suggests that levels of depression and anxiety increased in this time (Jung et al., 2020), and we do not know how this might have influenced the associations between well-being and psychological distress. Therefore, replication of the results is required.

## Conclusion

To our knowledge, this is the first study validating the dual continua model of mental health by explicitly examining the relationship between psychological distress and mental well-being within people. Our findings suggest that, at the group level, the dual continua model also seems to hold when examining the association between psychological distress and mental well-being within individuals. Yet, we also found that the association between distress and mental well-being can be substantially different for individuals, providing preliminary evidence that the assumption of the dual continua model might not be applicable for everyone. This study is an important step towards validating the applicability and universality of this widely used model, as it shows that the dual continua model in general also seems to hold on the level it is actually about, namely within individual people.

## Declarations

The data that support the findings of this study will be made openly accessible in a data repository after publication of this manuscript (https://osf.io/2srgu/).

## References

[CR1] Bolger N, Laurenceau JP (2013). Intensive longitudinal methods: An introduction to diary and experience sampling research.

[CR2] Cohen J (1988). Statistical power analysis for the behavioral sciences.

[CR3] Collins LM (2006). Analysis of longitudinal data: The integration of theoretical model, temporal design, and statistical model. Annual Review of Psychology.

[CR4] Conner TS, Lehman BJ, Mehl MR, Conner TS (2012). Getting started: Launching a study in daily life. Handbook of research methods for studying daily life.

[CR5] Curran PJ, Bauer DJ (2011). The disaggregation of within-person and between-person effects in longitudinal models of change. Annual Review of Psychology.

[CR6] De Vos JA, Radstaak M, Bohlmeijer ET, Westerhof GJ (2018). Having an eating disorder and still being able to flourish? Examination of pathological symptoms and well-being as two continua of mental health in a clinical sample. Frontiers in Psychology.

[CR7] Dejonckheere E, Erbas Y, Myin-Germeys I, Kuppens PE (2021). Designing an Experince sampling study. The open handbook of experience sampling methodology.

[CR8] Diener E, Ryan K (2009). Subjective well-being: A general overview. South Africa Journal of Psychology.

[CR9] Diener E, Suh EM, Lucas RE, Smith HL (1999). Subjective well-being: Three decades of progress. Psychological Bulletin.

[CR10] Drapeau A, Marchand A, Beaulieu-Prévost D, L’Abate L (2012). Epidemiology of psychological distress. Mental illnesses-understanding, prediction and control.

[CR11] Ebner-Priemer UW, Trull TJ (2009). Ecological momentary assessment of mood disorders and mood dysregulation. Psychological Assessment.

[CR12] Ebner-Priemer UW, Eid M, Kleindienst N, Stabenow S, Trull TJ (2009). Analytic strategies for understanding affective (in) stability and other dynamic processes in psychopathology. Journal of Abnormal Psychology.

[CR13] Etikan I, Musa SA, Alkassim RS (2016). Comparison of convenience sampling and purposive sampling. American Journal of Theoretical and Applied Statistics.

[CR14] Fat LN, Scholes S, Boniface S, Mindell J, Stewart-Brown S (2017). Evaluating and establishing national norms for mental wellbeing using the short Warwick–Edinburgh mental well-being scale (SWEMWBS): Findings from the health survey for England. Quality of Life Research.

[CR15] Franken K, Lamers SM, ten Klooster PM, Bohlmeijer ET, Westerhof GJ (2018). Validation of the mental health continuum-short form and the dual continua model of well-being and psychopathology in an adult mental health setting. Journal of Clinical Psychology.

[CR16] Gabriel AS, Podsakoff NP, Beal DJ, Scott BA, Sonnentag S, Trougakos JP, Butts MM (2019). Experience sampling methods: A discussion of critical trends and considerations for scholarly advancement. Organizational Research Methods.

[CR17] Grant F, Guille C, Sen S (2013). Well-being and the risk of depression under stress. PLoS One.

[CR18] Griffiths S, Stefanovski A (2019). Thinspiration and fitspiration in everyday life: An experience sampling study. Body Image.

[CR19] Gross JJ (1998). The emerging field of emotion regulation: An integrative review. Review of General Psychology.

[CR20] Hamaker EL, Mehl MR, Conner TS (2012). Why researchers should think" within-person": A paradigmatic rationale. Handbook of research methods for studying daily life.

[CR21] Hamaker EL, Nesselroade JR, Molenaar PC (2007). The integrated trait–state model. Journal of Research in Personality.

[CR22] Hamaker E, Ceulemans E, Grasman R, Tuerlinckx F (2015). Modeling affect dynamics: State of the art and future challenges. Emotion Review.

[CR23] Hamaker EL, Asparouhov T, Brose A, Schmiedek F, Muthén B (2018). At the frontiers of modeling intensive longitudinal data: Dynamic structural equation models for the affective measurements from the COGITO study. Multivariate Behavioral Research.

[CR24] Haver A, Akerjordet K, Caputi P, Furunes T, Magee C (2015). Measuring mental well-being: A validation of the short Warwick–Edinburgh mental well-being scale in Norwegian and Swedish. Scandinavian Journal of Public Health.

[CR25] Hoffman L (2007). Multilevel models for examining individual differences in within-person variation and covariation over time. Multivariate Behavioral Research.

[CR26] Hoffman L, Stawski RS (2009). Persons as contexts: Evaluating between-person and within-person effects in longitudinal analysis. Research in Human Development.

[CR27] Huang Z, Kohler IV, Kämpfen F (2020). A single-item visual analogue scale (VAS) measure for assessing depression among college students. Community Mental Health Journal.

[CR28] Huppert, F. A., & Whittington, J. E. (2003). Evidence for the independence of positive and negative well‐being: Implications for quality of life assessment. *British Journal of Health Psychology, 8*(1), 107–122. 10.1348/135910703762879246 10.1348/135910703762879246 12643820

[CR29] Iasiello, M., & Van Agteren, J. (2020). Mental health and/or mental illness: A scoping review of the evidence and implications of the dual-continua model of mental health *Evidence Base: A Journal of Evidence Reviews in Key Policy Areas,* (1), 1–45. 10.21307/eb-2020-001.

[CR30] Iasiello M, van Agteren J, Keyes CL, Cochrane EM (2019). Positive mental health as a predictor of recovery from mental illness. Journal of Affective Disorders.

[CR31] Jörngården A, Wettergen L, von Essen L (2006). Measuring health-related quality of life in adolescents and young adults: Swedish normative data for the SF-36 and the HADS, and the influence of age, gender, and method of administration. Health and Quality of Life Outcomes.

[CR32] Jung, S., Kneer, J., & Kruger, T. H. (2020). The German COVID-19 survey on mental health: Primary results. *MedRxiv.*10.1101/2020.05.06.20090340

[CR33] Keyes, C. L. (2002). The mental health continuum: From languishing to flourishing in life. *Journal of Health and Social Behavior*, 207–222. 10.2307/3090197. 12096700

[CR34] Keyes CL (2005). Mental illness and/or mental health? Investigating axioms of the complete state model of health. Journal of Consulting and Clinical Psychology.

[CR35] Keyes CL, Wissing M, Potgieter JP, Temane M, Kruger A, Van Rooy S (2008). Evaluation of the mental health continuum–short form (MHC–SF) in setswana-speaking south Africans. Clinical Psychology & Psychotherapy.

[CR36] Kievit R, Frankenhuis WE, Waldorp L, Borsboom D (2013). Simpson's paradox in psychological science: A practical guide. Frontiers in Psychology.

[CR37] Koval P, Kuppens P (2012). Changing emotion dynamics: Individual differences in the effect of anticipatory social stress on emotional inertia. Emotion.

[CR38] Kraiss JT, ten Klooster PM, Moskowitz JT, Bohlmeijer ET (2020). The relationship between emotion regulation and well-being in patients with mental disorders: A meta-analysis. Comprehensive Psychiatry.

[CR39] Lamers SMA, Westerhof GJ, Glas CAW, Bohlmeijer ET (2015). The bidirectional relation between positive mental health and psychopathology in a longitudinal representative panel study. The Journal of Positive Psychology.

[CR40] Larson R, Csikszentmihalyi M (2014). The experience sampling method. Flow and the foundations of positive psychology.

[CR41] Maes IH, Delespaul PA, Peters ML, White MP, van Horn Y, Schruers K, Anteunis L, Joore M (2015). Measuring health-related quality of life by experiences: The experience sampling method. Value in Health.

[CR42] Molenaar PC (2004). A manifesto on psychology as idiographic science: Bringing the person back into scientific psychology, this time forever. Measurement.

[CR43] Molenaar, P., & Newell, K. M. (2010). Individual pathways of change: Statistical models for analyzing learning and development. *American Psychological Association.*10.1037/12140-000

[CR44] Nakagawa S, Schielzeth H (2013). A general and simple method for obtaining R2 from generalized linear mixed-effects models. Methods in Ecology and Evolution.

[CR45] Nisenbaum R, Links PS, Eynan R, Heisel MJ (2010). Variability and predictors of negative mood intensity in patients with borderline personality disorder and recurrent suicidal behavior: Multilevel analyses applied to experience sampling methodology. Journal of Abnormal Psychology.

[CR46] Palmier-Claus J, Haddock G, Varese F (2019). Experience sampling in mental health research.

[CR47] Pinheiro, J., Bates, D., DebRoy, S., Sarkar, D., Heisterkamp, S., Van Willigen, B., & Maintainer, R. (2020). Nlme: Linear and nonlinear mixed effects models (version 3.1-151). https://CRAN.R-project.org/package=nlme

[CR48] R Core Team (2020). *R: A language and environment for statistical computing*. Vienna, Austria. R Foundation for Statistical Computing. https://www.R-project.org/

[CR49] Raudenbush SW, Collins LM, Sayer AG (2001). Toward a coherent framework for comparing trajectories of individual change. New methods for the analysis of change.

[CR50] Rauthmann JF, Horstmann KT, Sherman RA (2019). Do self-reported traits and aggregated states capture the same thing? A nomological perspective on trait-state homomorphy. Social Psychological and Personality Science.

[CR51] Ryff CD (1989). Happiness is everything, or is it? Explorations on the meaning of psychological well-being. Journal of Personality and Social Psychology.

[CR52] Ryff CD (2014). Self-realisation and meaning making in the face of adversity: A eudaimonic approach to human resilience. Journal of Psychology in Africa.

[CR53] Ryff CD, Keyes CLM (1995). The structure of psychological well-being revisited. Journal of Personality and Social Psychology.

[CR54] Schotanus-Dijkstra M, Drossaert CHC, Pieterse ME, Boon B, Walburg JA, Bohlmeijer ET (2017). An early intervention to promote well-being and flourishing and reduce anxiety and depression: A randomized controlled trial. Internet Interventions.

[CR55] Schotanus-Dijkstra M, Ten Have M, Lamers S, de Graaf R, Bohlmeijer ET (2017). The longitudinal relationship between flourishing mental health and incident mood, anxiety and substance use disorders. European Journal of Public Health.

[CR56] Schotanus-Dijkstra M, Keyes CLM, de Graaf R, ten Have M (2019). Recovery from mood and anxiety disorders: The influence of positive mental health. Journal of Affective Disorders.

[CR57] Schuurman NK, Ferrer E, de Boer-Sonnenschein M, Hamaker EL (2016). How to compare cross-lagged associations in a multilevel autoregressive model. Psychological Methods.

[CR58] Scollon CN, Kim-Prieto C, Diener E (2003). Experience sampling: Promises and pitfalls, strengths and weaknesses. Journal of Happiness Studies.

[CR59] Senker, K., Fries, S., & Grund, A. (2020). Mindfulness in everyday life: Between-and within-person relationships to motivational conflicts. *Current Psychology, 1-16*. 10.1007/s12144-020-00760-x

[CR60] Shiffman S, Stone AA, Hufford MR (2008). Ecological momentary assessment. Annual Review of Clinical Psychology.

[CR61] Stewart-Brown S, Tennant A, Tennant R, Platt S, Parkinson J, Weich S (2009). Internal construct validity of the Warwick-Edinburgh mental well-being scale (WEMWBS): A Rasch analysis using data from the Scottish health education population survey. Health and Quality of Life Outcomes.

[CR62] Stieger, S., Lewetz, D., & Swami, V. (2021). Emotional well-being under conditions of lockdown: An experience sampling study in Austria during the COVID-19 pandemic. *Journal of Happiness Studies, 1-18*. 10.1007/s10902-020-00337-2 10.1007/s10902-020-00337-2 PMC7778412 33424431

[CR63] Studer R (2012). Does it matter how happiness is measured? Evidence from a randomized controlled experiment. Journal of Economic and Social Measurement.

[CR64] Trompetter H, Lamers S, Westerhof GJ, Fledderus M, Bohlmeijer ET (2017). Both positive mental health and psychopathology should be monitored in psychotherapy: Confirmation for the dual-factor model in acceptance and commitment therapy. Behaviour Research and Therapy.

[CR65] Vaingankar JA, Abdin E, Chong SA, Sambasivam R, Seow E, Jeyagurunathan A, Picco L, Stewart-Brown S, Subramaniam M (2017). Psychometric properties of the short Warwick Edinburgh mental well-being scale (SWEMWBS) in service users with schizophrenia, depression and anxiety spectrum disorders. Health and Quality of Life Outcomes.

[CR66] Van Berkel N, Ferreira D, Kostakos V (2017). The experience sampling method on mobile devices. ACM Computing Surveys.

[CR67] Van de Pol M, Wright J (2009). A simple method for distinguishing within-versus between-subject effects using mixed models. Animal Behaviour.

[CR68] Versluis A, Verkuil B, Lane RD, Hagemann D, Thayer JF, Brosschot JF (2021). Ecological momentary assessment of emotional awareness: Preliminary evaluation of psychometric properties. Current Psychology.

[CR69] Viertiö S, Kiviruusu O, Piirtola M, Kaprio J, Korhonen T, Marttunen M, Suvisaari J (2021). Factors contributing to psychological distress in the working population, with a special reference to gender difference. BMC Public Health.

[CR70] Wang LP, Maxwell SE (2015). On disaggregating between-person and within-person effects with longitudinal data using multilevel models. Psychological Methods.

[CR71] Wang L, Zhang Q, Maxwell SE, Bergeman C (2019). On standardizing within-person effects: Potential problems of global standardization. Multivariate Behavioral Research.

[CR72] Westerhof, G. J. (2016). The dual continua model of mental health and illness: Theory, findings, and applications in psychogerontology. In L. Riby (Ed.), *handbook of gerontology research methods* (pp. 79-94). 10.4324/9781315771533-14

[CR73] Westerhof GJ, Keyes CL (2010). Mental illness and mental health: The two continua model across the lifespan. Journal of Adult Development.

[CR74] Wheeler L, Reis HT (1991). Self-recording of everyday life events: Origins, types, and uses. Journal of Personality.

[CR75] Williams VS, Morlock RJ, Feltner D (2010). Psychometric evaluation of a visual analog scale for the assessment of anxiety. Health and Quality of Life Outcomes.

[CR76] Wood AM, Joseph S (2010). The absence of positive psychological (eudemonic) well-being as a risk factor for depression: A ten year cohort study. Journal of Affective Disorders.

[CR77] Yearick, K. A. (2017). *Experience sampling methods (ESM) in organizations: a review* http://hdl.handle.net/2142/97635

[CR78] Zigmond AS, Snaith RP (1983). The hospital anxiety and depression scale. Acta Psychiatrica Scandinavica.

